# A Whole-Body Coordinated Motion Control Method for Highly Redundant Degrees of Freedom Mobile Humanoid Robots

**DOI:** 10.3390/biomimetics9120766

**Published:** 2024-12-16

**Authors:** Hao Niu, Xin Zhao, Hongzhe Jin, Xiuli Zhang

**Affiliations:** 1 School of Mechanical, Electronic and Control Engineering, Beijing Jiaotong University, Beijing 100044, China; 22110404@bjtu.edu.cn (H.N.); 23126175@bjtu.edu.cn (X.Z.); 2 The State Key Laboratory of Robotics and System, School of Mechatronics Engineering, Harbin Institute of Technology, Harbin 150001, China; hongzhejin@hit.edu.cn

**Keywords:** mobile humanoid robot, humanoid motion, whole-body motion control, dynamic movement primitives

## Abstract

Humanoid robots are becoming a global research focus. Due to the limitations of bipedal walking technology, mobile humanoid robots equipped with a wheeled chassis and dual arms have emerged as the most suitable configuration for performing complex tasks in factory or home environments. To address the high redundancy issue arising from the wheeled chassis and dual-arm design of mobile humanoid robots, this study proposes a whole-body coordinated motion control algorithm based on arm potential energy optimization. By constructing a gravity potential energy model for the arms and a virtual torsional spring elastic potential energy model with the shoulder-wrist line as the rotation axis, we establish an optimization index function for the arms. A neural network with variable stiffness is introduced to fit the virtual torsional spring, representing the stiffness variation trend of the human arm. Additionally, a posture mapping method is employed to map the human arm potential energy model to the robot, enabling realistic humanoid movements. Combining task-space and joint-space planning algorithms, we designed experiments for single-arm manipulation, independent object retrieval, and dual-arm carrying in a simulation of a 23-degree-of-freedom mobile humanoid robot. The results validate the effectiveness of this approach, demonstrating smooth motion, the ability to maintain a low potential energy state, and conformity to the operational characteristics of the human arm.

## 1. Introduction

In recent years, unmanned devices such as vehicles [1,2] and robots have rapidly developed, demonstrating great potential in many fields, particularly in the area of humanoid robotics. Humanoid robots are characterized by multiple degrees of freedom, a human-like appearance, and high flexibility [3]. In recent years, mobile humanoid robots that integrate arms and mobile platforms have begun to perform everyday tasks for humans. We envision such robots not only replacing humans in certain tasks but also engaging in interactive activities with humans, such as tidying a room or setting a table. When robots perform tasks or interact with humans, people generally prefer actions that resemble their own behavior patterns [4]. Consequently, human structure and movement have long been central research topics in physiology, anatomy, biomechanics, and neuroscience. To enable intuitive human prediction and understanding of robot behavior, mobile robots for manipulation need not only structural similarities to the human body but also dynamic behaviors that align with human movement patterns [5]. Based on these objectives, motion planning often begins with analyzing human movement, focusing on the characteristics of human motion during different tasks and applying these findings to the robot’s control strategy.

In traditional mobile manipulation robots, humanoid motion is primarily expressed through the movements of robotic arms. Researchers mainly approach this by examining human arm postures and motion trajectories, exploring the principles of human movement, and combining these insights with human kinematics and physiology to determine the corresponding motion trajectories for the robot. Control of humanoid robotic arms, achieved by mapping human arm postures onto the robotic arm to enable it to perform fine and complex tasks like a human arm, has become one of the most prominent applications in this field [6]. For example, researchers have identified characteristic patterns in upper limb movements, such as bell-shaped velocity curves [7], bell-shaped position variance [8], Fitts’ law, and time distribution [9]. These patterns are used to analyze joint velocity, acceleration, or trajectory during natural upper limb movements and serve as standards for generating humanoid motions in robotic arms. However, these studies primarily focus on the kinematic properties of upper limb movements, with limited exploration of other movement patterns and their impact on motion generation. To address this limitation, some researchers have proposed humanoid motion generation methods based on energy optimization and physiological models. For instance, Zhao et al. [10] guided the generation of humanoid trajectories by minimizing potential energy during robotic arm movement. Li et al. [11] determined humanoid arm postures by minimizing muscle strength, using this as a standard for generating humanoid trajectories. Qu et al. [12] achieved humanoid coordinated motion for dual arms by analyzing human arm movement patterns to obtain variable dual-arm coordination constraints. Yang et al. [13] estimated the stiffness characteristics of human arm endpoints by monitoring upper limb muscle activity and mapped these characteristics onto the robot, enabling it to learn and replicate human motion and stiffness features. Chen et al. [14] designed a multimodal sensor system capable of capturing and precisely controlling human upper limb movements, thereby realizing humanoid control of robotic arms.The above approaches to exploring principles of human motion primarily focus on robotic arms. While these methods can generate humanoid-like motion trajectories for specific tasks, most studies remain limited to task-specific humanoid motion planning and rarely address mobile manipulation. Additionally, these studies tend to use singular humanoid variables and lack flexibility in parameter selection, which may lead to suboptimal performance in complex, multi-task environments.

The various numbers of joints and their different configurations in mobile humanoid robots create a wide range of possible movement states, resulting in highly complex motion with diverse state variations during operation. With the continuous development of intelligent learning methods, research on humanoid motion for robotic arms has also made further progress. Duarte et al. [15] achieved humanoid motion control for robots by designing various motion states and modeling them with Gaussian Mixture Models (GMMs). Chang et al. [16] used Gaussian Mixture Regression (GMR) to construct continuous task parameter models that align with characteristic movements, proposing a humanoid motion generation method with smooth trajectories. Kuo et al. [17] introduced a motion controller based on machine learning and fuzzy control, applying the Deep Deterministic Policy Gradient (DDPG) algorithm to enable humanoid robots to autonomously plan arm movements and joint angles. Pignat and Calinon [18] used a hidden semi-Markov model, allowing robots to assist humans in completing complex tasks such as dressing. Yi et al. [19] developed an autonomous robotic grasping system using K-means clustering and dynamic movement primitives, capable of performing precise operations through various machine learning methods. Yang et al. [20] utilized reinforcement learning algorithms based on multiple features of human upper limb movements to plan humanoid motions for robotic arms, achieving natural and smooth movement behavior. While these methods enable humanoid manipulation of arms, controlling mobile manipulation robots requires not only humanoid arm movements but also coordinated motions involving the chassis, arms, and torso. Although humanoid motion planning methods combined with machine learning have significantly improved the accuracy and efficiency of robot motion, these approaches still face challenges. First, limited training or demonstration data can restrict model generalization. Additionally, these methods often require carefully crafted reward functions to ensure the model learns appropriate movement strategies. Training can be time-consuming, and the resulting data models frequently lack comprehensive coverage of the complex motion patterns of human arms, limiting the robustness and adaptability of the generated motions.

**Figure 1:**
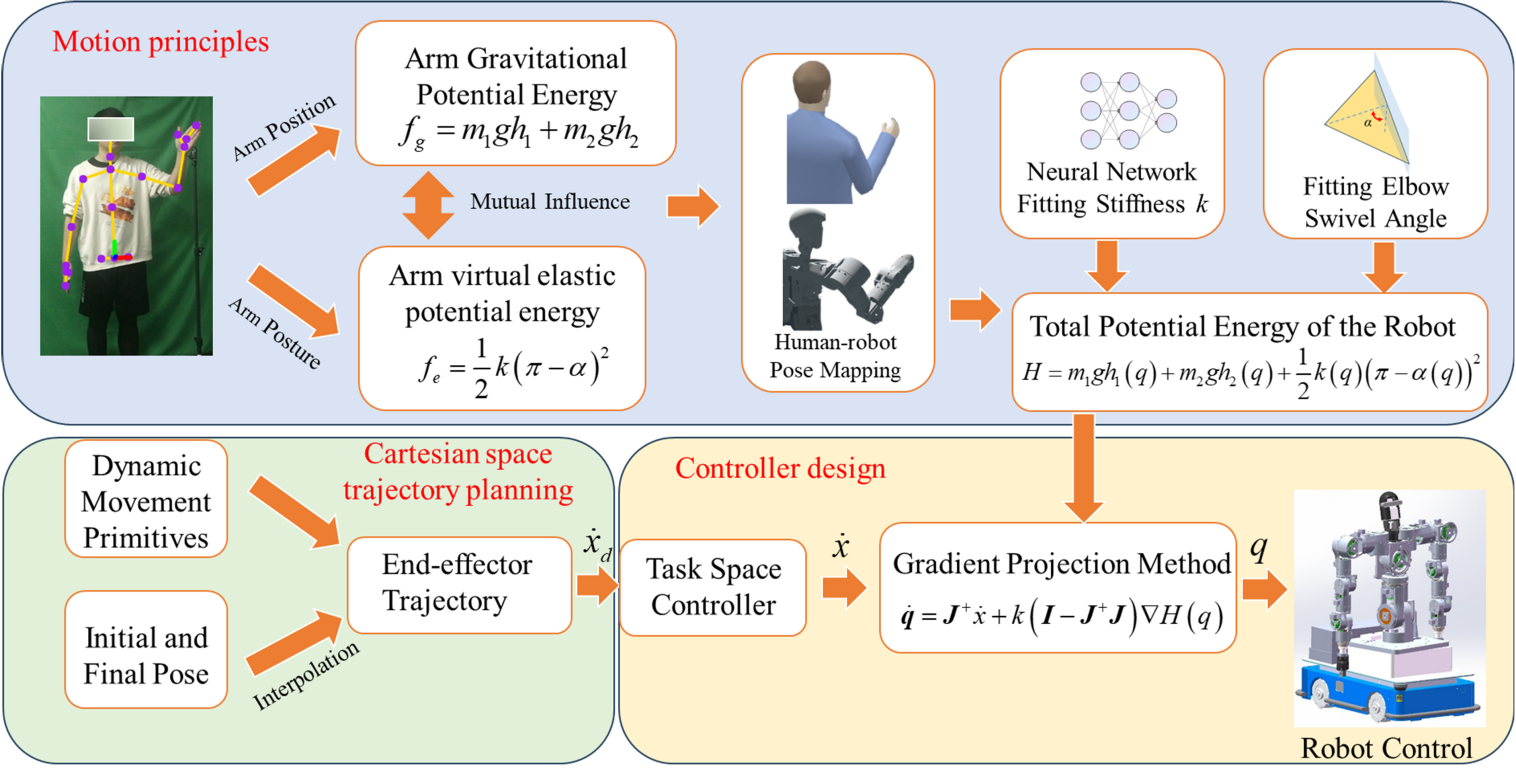
Whole -body coordinated motion control architecture for mobile humanoid robot.

Unlike previous studies focused primarily on humanoid motion planning for arms, this research develops a whole-body motion planning algorithm specifically for mobile humanoid robots. The overall approach of the paper is illustrated in Figure 1. By collecting data on human upper limb grasping movements and analyzing arm motion characteristics, we propose an arm motion planning algorithm that emulates the principles of human potential energy motion. Regarding the intrinsic motion mechanisms of the arm, we analyzed the changes in gravitational potential energy and the elastic potential energy of a virtual torsional spring during the grasping process and mapped these variations onto the robot. By using a neural network to fit the trend of virtual torsional spring stiffness changes, we achieve control over the arm’s movements. For end-effector task-space planning, we use Dynamic Movement Primitives (DMP) to generate motion trajectories. When both hands are cooperating, the task-space trajectory for the secondary arm is generated based on the trajectory of the primary arm. When the hands operate independently, each arm plans its own task-space trajectory using the DMP algorithm. Finally, we integrate the motion principles with task-space trajectories through the gradient projection method, generating joint-space trajectories for the mobile manipulation robot and achieving control of all 23 degrees of freedom. The effectiveness of this algorithm was validated through simulation experiments.

## 2. Extracting Motion Principles

**Figure 2:**
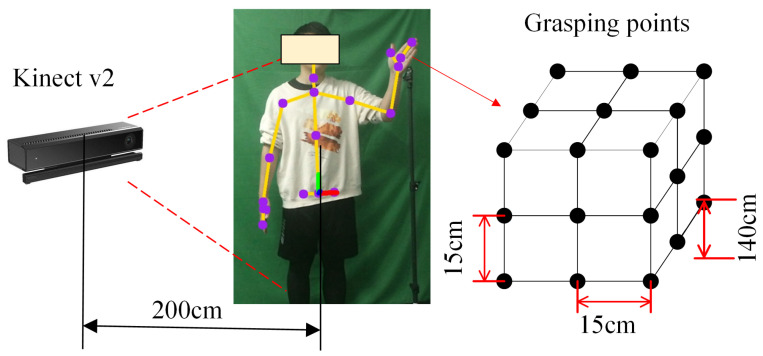
Illustration of grasping experiments at different heights.

The objective of this study is to achieve humanoid manipulation tasks for mobile humanoid robots by exploring the motion principles of arm movements. To this end, we first conducted a systematic investigation into the humanoid characteristics of arm motion. We designed 27 groups of grasping experiments at different heights and 9 groups of tabletop grasping experiments, using a Kinect sensor (Microsoft Corp., Redmond, WA, USA) to capture the 3D positions of key skeletal points in the arm throughout the grasping process in real time. Taking the waist joint position as the reference zero point, we analyzed the movement trajectory of the elbow joint and palm from a naturally hanging position to the target grasping position, focusing on the height variation characteristics under different grasping points. The grasping scene used in the experiment is shown in Figure 2. The Kinect camera was fixed 200 cm in front of the subject to capture the 3D position information of key points in the upper limb motion in real time, including the positions of the shoulder, elbow, and wrist joints. During the experiment, the subject was asked to sequentially move their hand to 27 preset target points. The target points were evenly distributed across three mutually parallel vertical planes, with each target point spaced 15 cm apart, and the lowest target point set at a height of 140 cm from the ground. Similarly, the tabletop grasping experiment was designed for the arm to sequentially grasp nine preset target points on the tabletop. During the experiment, the Kinect sensor was again used to capture the 3D position information of key points at the shoulder, elbow, and wrist while the subject performed the grasping tasks. The experiment design aims to systematically study arm movement characteristics under different height and position conditions, further uncovering the motion patterns involved in human grasping tasks, thereby providing a basis for the arm motion principles of mobile humanoid robots.

**Figure 3:**
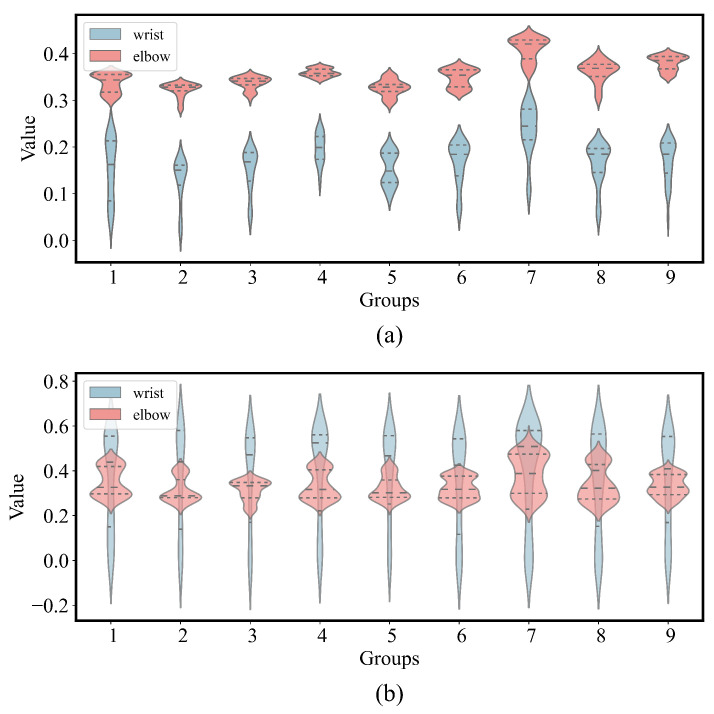
Violin plot of elbow and wrist height variation during arm movement. (**a**) Changes in wrist and elbow height in the desktop grasping experiment. (**a**) Changes in wrist and elbow height in the lift-arm experiment.

Using the tabletop grasping experiment and the nine grasping experiments on the lowest plane as examples, we created violin plots of the height variations for the elbow and wrist, as shown in Figure 3. This plot illustrates the positional variation characteristics of the elbow and wrist joints in the vertical direction during the experiment. The analysis results indicate that the elbow joint shows relatively minor vertical positional changes, while the wrist joint exhibits comparatively larger height variations. This difference arises from the elbow’s tendency to remain in a relatively low position throughout the movement, keeping the forearm in a lower position and, consequently, the arm in a state of lower gravitational potential energy. Additionally, the forearm’s low position allows for greater movement space and flexibility, allowing most of the operational adjustments to be made by moving the forearm. Furthermore, the lower distribution of the elbow joint also results in a smaller overall arm angle, helping the arm maintain a more compact posture when performing grasping tasks.

The above grasping experiments suggest that arm movement is influenced by the arm’s position and overall posture. Assuming kinetic energy effects are negligible, we consider human arm energy as the sum of gravitational potential energy stored in the arm and elastic potential energy stored in the muscles. Based on this assumption, we treat these two types of potential energy as the core optimization indicators for arm movement, simulating the influence of arm position and posture on movement. In this simplified model, the arm’s gravitational potential energy considers only the upper arm and forearm (excluding the weight of the hand) and can be expressed in the following form:

(1) $$f_{g}=m_{1}gh_{1}+m_{2}gh_{2}$$

where $m_{1}$ and $m_{2}$ represent the masses of the upper arm and forearm, respectively, while $h_{1}$ and $h_{2}$ denote the heights of the upper arm and forearm. $h_{1}$ and $h_{2}$ are functions of the upper limb joint variables and dynamically change with joint angle variations. The symbol *g* represents gravitational acceleration.

**Figure 4:**
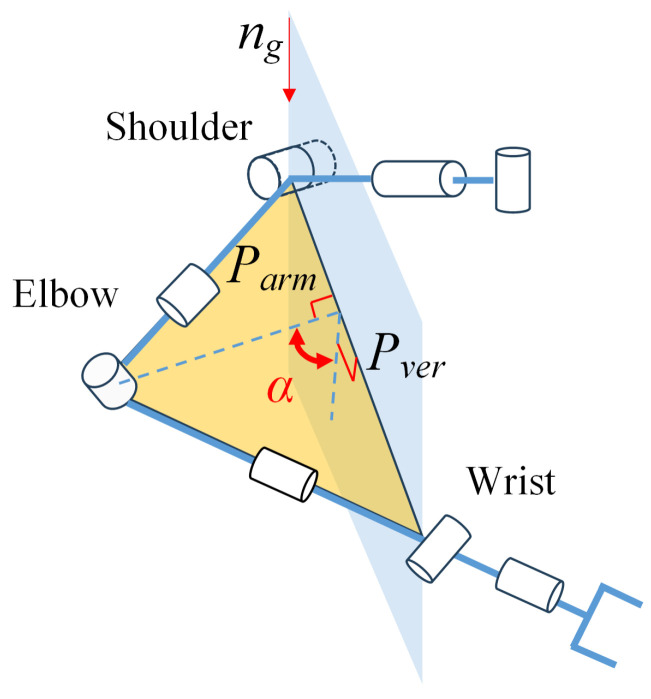
Illustration of arm angle.

Each arm of the mobile humanoid robot has 8 degrees of freedom. Unlike the traditional model that considers the human arm to have 7 degrees of freedom, this study includes the sternoclavicular joint, adding an additional degree of freedom to arm movement. For humanoid control of this redundant degree-of-freedom arm, a criterion is needed to generate appropriate arm postures. The generation of arm posture can be determined using the arm angle variable, ensuring effective utilization of redundancy and natural motion. We consider the change in arm angle caused by muscle action as similar to the effect produced by a torsional spring. Specifically, the line from the elbow to the wrist can be viewed as the rotational axis of this virtual torsional spring, and the angle α between the plane formed by the upper arm and forearm and the vertical plane is defined as the arm angle, as illustrated in Figure 4. Thus, the virtual elastic potential energy of the arm can be expressed as:

(2) $$f_{e}=\frac{1}{2}k{\left(\pi -\alpha \right)}^{2}$$

where *k* represents the stiffness of the virtual torsional spring, and α represents the arm angle.

The total potential energy of the arm is the sum of the gravitational potential energy and the virtual elastic potential energy, expressed as:

(3) $$f_{p}=f_{g}+f_{e}=m_{1}gh_{1}+m_{2}gh_{2}+\frac{1}{2}k{\left(\pi -\alpha \right)}^{2}$$

The principle of minimum total potential energy states that an object or structure tends toward a position of minimum total potential energy, achieving a stable equilibrium configuration. Based on this principle, we assume that the total potential energy of the human arm is also minimized when performing an action. From this, we can derive the parameter values corresponding to this equilibrium state, including the calculation formula for the stiffness *k* of the virtual torsional spring:

(4) $$k=\frac{{\dot{f}}_{g}}{\pi -\alpha }$$

The above process primarily calculates the virtual stiffness of the human arm, rather than the virtual spring stiffness of a robotic arm. Therefore, to effectively transfer this principle to a robotic system, structural differences between the two must be considered. Due to significant differences in mass distribution and dimensional configuration between the robotic and human arms, the stiffness model of the human arm cannot be directly applied to the robot. According to studies in the literature [21], a posture mapping method can be introduced to map human arm postures to robotic arm postures. However, this mapping process may cause shifts in the position of the robot’s elbow and wrist, and there may also be discrepancies between the robot’s arm angle and the human arm angle. Consequently, the virtual torsional spring stiffness *k* value for the robotic arm must be re-derived.

**Figure 5:**
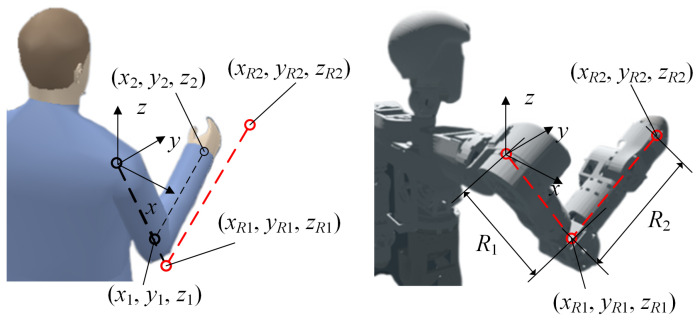
Human-robot posture mapping process.

The mapping process is illustrated in Figure 5. Specifically, during human arm movement, we scale the human shoulder-to-elbow and elbow-to-wrist vectors proportionally according to the lengths of the robot’s upper arm and forearm. The mapped shoulder-to-elbow and elbow-to-wrist vectors for the robot can be expressed as:

(5) $$\vec{SE_{R}}=\frac{\bar{SE}}{∥SE∥}∥SE_{R}∥$$

(6) $$\vec{E_{R}W_{R}}=\frac{\bar{EW}}{∥EW∥}∥E_{R}W_{R}∥$$

where $\vec{SE_{R}}$ and $\vec{E_{R}W_{R}}$ represent the mapped shoulder-elbow vector and elbow-wrist vector of the robot. $\bar{SE}$ and $\bar{EW}$ are the shoulder-elbow vector and elbow-wrist vector of the human body, respectively. ∥SE∥ and ∥EW∥ represent the lengths of the upper arm and forearm of the human body, while $∥SE_{R}∥$ and $∥E_{R}W_{R}∥$ represent the lengths of the upper arm and forearm of the robot, respectively.

Through the mapping process described above, we can derive the corresponding positions of the robot’s elbow and wrist joints for a given human posture and subsequently calculate the robot’s arm angle. Similar to the calculation process for the human arm’s virtual torsional spring stiffness, we can use the mapped arm angle and joint positions to derive the virtual torsional spring stiffness *k* for the robot arm. Our goal is to establish a mapping relationship between the virtual torsional spring stiffness and the wrist position, and further, using the forward kinematics model of the robot wrist position, to express the relationship between the virtual stiffness *k* and the joint variables *q*. At this point, the potential energy term can be represented as:

(7) $$f_{p}=f_{g}+f_{e}=m_{1}gh_{1}+m_{2}gh_{2}+k\left(x,y,z\right){\left(\pi -\alpha \right)}^{2}/2$$

Given the unknown numerical relationship between the virtual torsional spring stiffness *k* and the wrist position, we construct a mapping model using a neural network. When handling nonlinear data, we chose Support Vector Regression (SVR) as a comparison to the neural network. The coefficient of determination $R^{2}=0.9304$ for the neural network on the test set is higher than that of support vector regression, which is $R^{2}=0.9169$. Additionally, the mean squared error (MSE) of the neural network is 0.1673, which is lower than the MSE of the SVR model at 0.2352. We consider this output error to be acceptable. After comparison, the current neural network structure is reasonable and can effectively fit the relationship between the stiffness output of the virtual torsion spring and the input of the wrist position.

In constructing the dataset, we collected data from 27 groups of grasping actions at different heights and 9 groups of tabletop grasping actions at different positions, for a total of 36 groups of data. After the human posture data were processed through a posture mapping, the corresponding robot virtual stiffness was calculated, resulting in 36 sets of robot data. These 36 sets of data were integrated, yielding approximately 5000 data records. From this, 300 records were selected as the validation set, and the remaining data were used as the training set for neural network training. We ensure that the training data and testing data are independent. Both the training and testing data are derived from the robot’s virtual stiffness results calculated from the human body data mapping. However, the data in the testing set was not seen during the training process.Through a series of experiments, we observed that as the dataset size increased, the accuracy of the neural network training improved. However, there is a diminishing return effect: after reaching a certain amount of data, the improvement in accuracy starts to flatten out. Specifically, after approximately 3000 data records, the model’s performance stabilizes, but larger datasets (such as those beyond 3000 records) can further reduce the risk of overfitting. During the data preprocessing stage, we applied noise filtering and removed unreasonable outliers to ensure the quality of the training data, reducing the interference of these anomalies on the model. Currently, we are using a total of approximately 5000 data records, with 300 data points allocated as a validation set to test the model’s performance. The mean squared error (MSE) on the test set is 0.1673. Considering the quality of the collected data, we find the current MSE result acceptable. At the same time, we believe that the diversity of the data and its coverage across the workspace are more important than the sheer volume of data.

**Figure 6:**
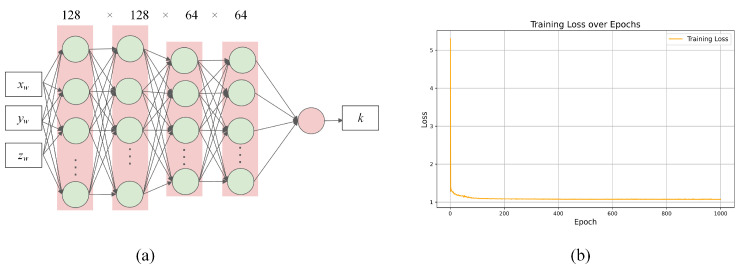
Neural network for virtual stiffness fitting. (**a**) Neural network architecture used for virtual torsion spring fitting process. (**b**) Loss variation during training process.

As shown in Figure 6a, we designed a fully connected neural network with the wrist’s spatial position coordinates $\left(x_{w},y_{w},z_{w}\right)$ as inputs and the corresponding virtual torsional spring stiffness *k* of the robot as the output. The neural network is implemented using the PyTorch framework, with the Mean Squared Error (MSE) as the loss function. During training, the learning rate is set to 0.01, the batch size is 32, and training is conducted for 1000 epochs using the Adam optimizer. The training loss curve is shown in Figure 6b. This network’s mapping relationship enables nonlinear fitting of the *k* values. Furthermore, by combining the wrist’s forward kinematics model, we can derive the relationship between the virtual torsional spring stiffness *k* and the joint variables *q*, yielding an expression for the virtual torsional spring stiffness as a function of joint variables, k(q).

After obtaining the virtual torsional spring stiffness k(q), it is also necessary to express the arm angle α as a function of the joint variables *q*. To achieve this, we use the relationship between the arm angle and wrist pose from [22] as an intermediate step to establish a mapping between the arm angle α and joint variables *q*. Specifically, the relationship between the arm angle and wrist pose can be represented by the following mathematical expression:

(8) $$\begin{array}{cc} \alpha = & 0.88\cos\left(x_{5}+2.30\right)+0.49\cos\left(x_{5}+x_{6}+2.98\right)\\ \; & -0.21\mathrm{atan}2\left(x_{3},x_{2}\right)-0.64\sin\left(x_{4}\right)-1.39-0.50\mathrm{atan}2\left(x_{2},x_{1}\right)\\ \; & -0.22\cos\left(x_{4}+x_{5}+x_{6}\right)+0.47\cos\left(x_{6}+0.60\right)-0.15\mathrm{atan}2\left(x_{3},x_{1}\right)\\ \; & +0.34\cos\left(x_{4}+x_{6}+1.07\right)-0.40\cos\left(x_{4}+x_{5}\right) \end{array}$$

where $x_{1}$, $x_{2}$, and $x_{3}$ represent the *X*, *Y*, and *Z* position components of the wrist pose, while $x_{4}$, $x_{5}$, and $x_{6}$ correspond to the ZYZ Euler angles. Since both the position and orientation of the wrist are functions of the joint variables q, we can further derive an expression for the relationship between the arm angle α and the joint variables *q*. Based on this relationship, the potential energy term of the robotic arm can be expressed as a function of the joint variables *q*, enabling a quantitative description of the relationship between the potential energy and joint variables:

(9) $$f_{p}=f_{g}+f_{e}=m_{1}gh_{1}\left(q\right)+m_{2}gh_{2}\left(q\right)+k\left(q\right){\left(\pi -\alpha \left(q\right)\right)}^{2}/2$$

Building on this, we introduce an auxiliary optimization term to further enhance the arm’s performance during the robot’s movement. We aim for each joint to stay as close as possible to the center of its range of motion during operation, avoiding extreme positions to reduce instability and mechanical stress associated with nearing joint limits, thereby improving overall maneuverability. The optimization objective function can be expressed as:

(10) $$H=w_{1}f_{p}\left(q\right)+w_{2}h_{1}\left(q\right)+w_{3}h_{2}\left(q\right)$$

where $w_{1}$ and $w_{3}$ are scalar coefficients, and $w_{2}$ is a coefficient matrix. The joint limit avoidance term can be expressed as:

(11) $$h_{1}=\sum_{i=1}^{19}\frac{{\left(q_{\max}\left[i\right]-q_{\min}\left[i\right]\right)}^{2}}{4\left(q_{\max}\left[i\right]-q\left[i\right]\right)\left(q\left[i\right]-q_{\min}\left[i\right]\right)}$$

The manipulability optimization term can be expressed as:

(12) $$h_{2}=\sqrt{\det\left(J\left(q\right)J^{T}\left(q\right)\right)}$$

## 3. Operational Space Trajectory Planning Algorithm

After constructing the corresponding optimization objective function, we aim to use the gradient projection method to generate the corresponding joint-space trajectory. To achieve this, it is first necessary to accurately plan the trajectory of the robot’s end-effector, ensuring that the end-effector trajectory meets task requirements while the joint movement trajectory adheres to the defined optimization criteria. When performing arm manipulation tasks, the robot needs to determine how to move its end-effector to complete the intended task. To generate adaptive trajectories, we introduce a Dynamic Movement Primitives (DMP) model based on demonstration learning. The DMP model, built on the ordinary differential equations of a spring-mass-damper system, captures and reproduces complex motion patterns. Its mathematical definition is as follows:

(13) $$\left\{\begin{array}{c} \tau \dot{v}=K\left(g-x\right)-Dv-K\left(g-x_{0}\right)s+Kf\left(s\right)\\ \tau \dot{x}=v \end{array}\right.$$

where vectors x and $v\in R^{d}$ represent the system’s position and velocity, respectively, and $x_{0}$ and $g\in R^{d}$ are the initial and target positions, respectively. The matrices K and D are the system’s stiffness and damping terms, respectively, both of which are diagonal matrices. Specifically, $K=\mathrm{diag}(K_{1},K_{2},\cdots ,K_{d})$ and $D=\mathrm{diag}(D_{1},D_{2},\cdots ,D_{d})$. The stiffness-damping relationship needs to satisfy $D_{i}=2\sqrt{K_{i}}$, and the system will have fast and smooth recovery characteristics. However, for relatively simple tasks, such as following a straight-line trajectory, we require higher stiffness while maintaining the stiffness-damping relationship. For more complex tasks, such as object manipulation or interaction with dynamic objects, choosing relatively lower stiffness and damping values can provide higher adaptability. Through continuous manual adjustments, we used K=1050 and the corresponding $D_{i}=2\sqrt{1050}$. The scalar τ is a time-scaling factor that allows the trajectory to be executed faster or slower. The function f is the forcing term. The function f is the forcing term. The scalar s∈(0,1] represents the re-parameterization of time t∈[0,T]. With this, the forward system control can be represented as:

(14) $$\tau \dot{s}=-\alpha s$$

where $\alpha \in R_{+}$, and the initial state is s(0)=1.

The forcing term $f\left(s\right)={\left[f_{1}\left(s\right),f_{2}\left(s\right),\cdots ,f_{d}\left(s\right)\right]}^{T}$ is represented by basis functions. Each component $f_{p}\left(s\right)$ for p=1,2,⋯,d is expressed as:

(15) $$f_{p}\left(s\right)=\frac{\sum_{i=0}^{N}\omega_{i}\psi_{i}\left(s\right)}{\sum_{i=0}^{N}\psi_{i}\left(s\right)}s$$

where $\omega_{i}\in R$ represents the weight, and $\psi_{i}\left(s\right)$ is a Gaussian basis function, defined as:

(16) $$\psi_{i}\left(s\right)=\exp\left(-h_{i}{\left(s-c_{i}\right)}^{2}\right)$$

The parameters $c_{i}$, $h_{i}$, and *N* are determined based on the task requirements.

At the same time, a corresponding repulsion term can be introduced into the Dynamic Movement Primitive (DMP) framework to achieve the effect of pushing the trajectory away from obstacles. In this case, the DMP framework becomes:

(17) $$\tau \dot{v}=K\left(g-x\right)-Dv-K\left(g-x_{0}\right)s+Kf\left(s\right)+\varphi \left(x,v\right)$$

The repulsion term φ is considered a function of the trajectory position x and velocity v. The commonly used method is the potential field approach. For a potential field that depends on position x and velocity v, the repulsion term is defined as the negative gradient with respect to position:

(18) $$\varphi \left(x,v\right)=-\nabla_{x}U\left(x,v\right)$$

where U(x,v) is the potential function. To achieve trajectory obstacle avoidance, we use the volumetric obstacle dynamic potential function proposed in [23]:

(19) $$U_{D}\left(x,v\right)=\left\{\begin{array}{cc} \lambda {\left(-\cos\theta \right)}^{\beta }\frac{∥v∥}{C^{\eta }\left(x\right)}, & \theta \in \left(\frac{\pi }{2},\pi \right]\\ 0, & \theta \in \left[0,\frac{\pi }{2}\right] \end{array}\right.$$

where λ, β, and η are constant gains, and angle θ is the angle between the trajectory’s velocity and the direction from the trajectory position to the closest point on the obstacle. C(x) is an equipotential function that vanishes on the surface of the obstacle:

(20) $$C\left(x\right)={\left(\frac{x_{1}}{f_{1}\left(x\right)}\right)}^{2n}+{\left(\frac{x_{2}}{f_{2}\left(x\right)}\right)}^{\frac{2m}{2n}}+{\left(\frac{x_{3}}{f_{3}\left(x\right)}\right)}^{2m}-1$$

By adjusting the parameters *m*, *n* and the functions $f_{1}$, $f_{2}$, $f_{3}$, it is possible to model obstacles of any shape.

The movement modes of a robot’s dual arms are not fixed and can be categorized into two types based on the kinematic coordination between the arms: independent dual-arm motion and coordinated dual-arm motion. In the independent motion mode, each robotic arm independently plans its own trajectory using the DMP model, without influencing the other. In the coordinated motion mode, however, both arms work together to accomplish a specific task. For example, when carrying an object, the two arms and the object form a closed-loop system with constraint conditions. In this system, the motion of each arm must satisfy these constraints to achieve stable carrying and precise manipulation of the object.

In the dual-arm robotic system, we designate one arm as the primary arm and the other as the secondary arm. The end-effector position trajectory $P_{M}$ of the primary arm is generated using the DMP model, while the end-effector orientation trajectory $R_{M}$ is calculated through interpolation between the initial orientation and the target end orientation. The transformation of the object’s coordinate frame relative to the world coordinate system can be determined either by the transformation matrix of the primary arm or that of the secondary arm. We establish a pose constraint equation between the dual arms and the object, expressed in the following form:

(21) $$T_{M_{T}}^{R_{W}}\cdot T_{O_{C}}^{M_{T}}=T_{S_{T}}^{R_{W}}\cdot T_{O_{C}}^{S_{T}}$$

where $T_{M_{T}}^{R_{W}}$ and $T_{S_{T}}^{R_{W}}$ represent the homogeneous transformation matrices of the end-effectors of the primary and secondary arms in the world coordinate system, respectively. $T_{O_{C}}^{M_{T}}$ and $T_{O_{C}}^{S_{T}}$ represent the homogeneous transformation matrices of the object being transported in the coordinate systems of the primary and secondary arms, respectively.

During the process of carrying an object, in addition to considering the pose constraint relationship between the end-effectors of the dual arms and the object, it is also necessary to introduce a velocity constraint relationship for the dual-arm tool coordinate systems. Specifically, the angular velocities of the dual-arm tool coordinate systems relative to the world coordinate system should satisfy the following relationship:

(22) $$V_{S_{T}}^{R_{W}}=V_{M_{T}}^{R_{W}}+T_{M_{T}}^{R_{W}}\cdot V_{S_{T}}^{M_{T}}$$

where $V_{S_{T}}^{R_{W}}$ and $V_{M_{T}}^{R_{W}}$ represent the linear velocities of the end-effectors of the secondary and primary arms in the global coordinate system, respectively. $V_{S_{T}}^{M_{T}}$ represents the linear velocity of the secondary arm’s tool frame relative to the primary arm’s tool frame.

## 4. Whole-Body Coordinated Controller

In Section 2 and Section 3 of the previous research, we planned the arm motion principles and the end-effector trajectory, respectively. Given that robotic arm motion requires high precision at the end-effector, we need to ensure accurate tracking of the end-effector. Therefore, the task-space control law is designed as follows:

(23) $$\dot{x}\left(t\right)={\dot{x}}_{d}\left(t\right)+K_{p}\left(x_{d}\left(t\right)-x\left(t\right)\right)+K_{i}\int_{0}^{t}\left(x_{d}\left(t\right)-x\left(t\right)\right)dt$$

where ${\dot{x}}_{d}\left(t\right)$ represents the planned end-effector velocity, $x_{d}\left(t\right)$ represents the planned end-effector position, and x(t) represents the actual position. $K_{p}$ and $K_{i}$ are gain coefficients.

The relationship between the velocity $\dot{x}$ of the robot’s end-effector and the velocities $\dot{q}$ of each joint is given by $\dot{x}=J\left(q\right)\dot{q}$, where J(q) represents the Jacobian matrix. Considering the motion characteristics of the entire mobile humanoid robot, we can further derive the following relationship:

(24) $$\left[\begin{array}{c} {\dot{X}}_{L}\\ {\dot{X}}_{R} \end{array}\right]=\left[\begin{array}{cccc} J_{\mathrm{mobile}} & J_{\mathrm{waist}} & J_{\mathrm{arm}\_1} & 0\\ J_{\mathrm{mobile}} & J_{\mathrm{waist}} & 0 & J_{\mathrm{arm}\_r} \end{array}\right]\left[\begin{array}{c} u\\ {\dot{q}}_{\mathrm{waist}}\\ {\dot{q}}_{\mathrm{arm}\_1}\\ {\dot{q}}_{\mathrm{arm}\_r} \end{array}\right]$$

where ${\dot{X}}_{L}\in R^{6}$ and ${\dot{X}}_{R}\in R^{6}$ represent the tool frame velocities of the left and right arms, respectively, obtained from task-space control, with the right arm assumed as the primary arm and the left arm as the secondary arm. The Jacobian matrix of the entire robot is $J\left(q\right)\in R^{12\times 23}$. The joint velocity $\dot{q}\in R^{23}$ includes the rotational velocities of the Mecanum wheels on the mobile base $u\in R^{4}$, the waist joint velocity ${\dot{q}}_{\mathrm{waist}}\in R^{3}$, and the joint velocities of the left and right arms ${\dot{q}}_{\mathrm{arm}\_1}\in R^{8}$ and ${\dot{q}}_{\mathrm{arm}\_r}\in R^{8}$, respectively.

Based on the null-space projection method, additional tasks can be projected into the null space of the Jacobian matrix. Then, the projected joint inputs can be superimposed to obtain the final joint control inputs. Given the velocity of the robot’s end-effector and incorporating the gradient information of the optimization objective function, the following joint-space velocity expression can be derived:

(25) $$\dot{q}=J^{+}\dot{x}+k\left(I-J^{+}J\right)\nabla H\left(q\right)$$

where $J^{+}\in R^{23\times 12}$ is the pseudo-inverse of the Jacobian matrix *J*, defined as $J^{+}=J^{T}{\left(JJ^{T}\right)}^{-1}$. $I\in R^{23\times 23}$ is the identity matrix, and *k* is a weighting factor that controls the influence of the gradient term. $\nabla H\left(q\right)=\partial H\left(q\right)/\partial q\in R^{23}$ represents the gradient of the optimization objective function H(q) in joint space *q*, indicating the direction of joint velocities that achieve the fastest change in H(q). Considering the complexity of such a robotic system, we did not derive the corresponding analytical expressions. Instead, we performed the numerical calculations of the Jacobian matrix using the Robotics Toolbox reinvented for the Python kinematics library [24], based on the relevant D-H kinematic parameters.

By projecting the gradient of the optimization objective into the null space of the Jacobian matrix, and leveraging the properties of the null space, we can ensure optimized control of additional objectives while accomplishing the primary task of following the robot arm’s end-effector velocity. This control strategy enables the construction of a whole-body coordinated control framework, allowing the robot to perform coordinated control of additional tasks across all 23 degrees of freedom.

## 5. Experiments and Results

**Figure 7:**
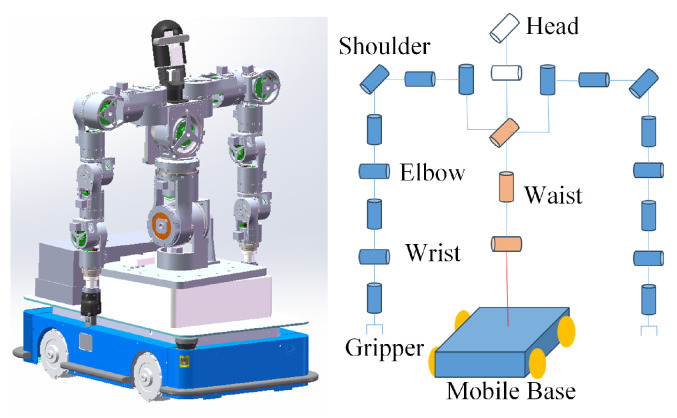
Mobile humanoid robot model.

The mobile humanoid robot system used in this study is shown in Figure 7. The system’s overall structure consists of two parts: a humanoid upper body and a mobile base. The robot’s mobile base is equipped with four Mecanum wheels, enabling omnidirectional movement. The humanoid upper body includes two arms, each with 8 degrees of freedom, a waist with 3 degrees of freedom, and a head with 2 degrees of freedom. In this study, we did not consider planning for the 2 degrees of freedom of the head. Unlike conventional seven-degree-of-freedom redundant robotic arms, the robot’s arms have an additional sternoclavicular joint at the shoulder. The remaining seven joints follow an unbiased Spherical-Rotational-Spherical (SRS) configuration, with symmetrical arm distribution. The kinematic parameters of the joints are shown in Table 1.

To apply the motion planning scheme proposed in the previous section to the mobile humanoid robot system and validate the algorithm’s accuracy and effectiveness, we designed the following three experiments:

A. Operational Space Task Trajectory Generalization and Generation Experiment: This experiment aims to verify whether the system can generate corresponding generalized and obstacle-avoidance trajectories based on reference trajectories.
B. Independent Single-Arm Motion Experiment for the Mobile Humanoid Robot: This experiment is designed to validate the effectiveness of the motion principles, specifically, whether the potential energy can be reduced during single-arm movement and if the movement can achieve a natural and smooth trajectory.
C. Coordinated Dual-Arm Motion Experiment for the Mobile Humanoid Robot: This experiment primarily verifies whether the end-effectors of both arms can cooperatively perform an object-grasping task according to the planned trajectories throughout the entire motion process.

**Figure 8:**
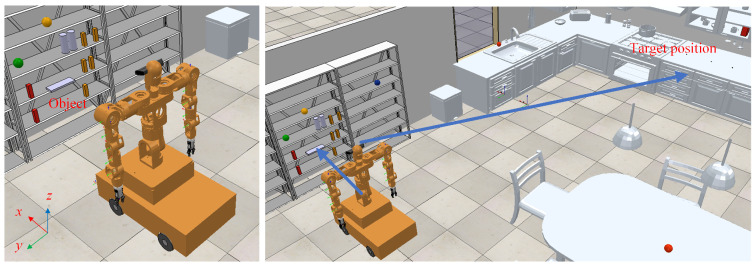
Experimental simulation scenario for the mobile humanoid robot.

The simulation environment uses CoppeliaSim, with the simulation scenario shown in Figure 8. The computing platform we are currently using is a personal laptop equipped with an Intel i7 CPU (16GB RAM) and an NVIDIA GeForce RTX 3060 GPU (NVIDIA Corporation, Santa Clara, CA, USA). We designed the robot to perform corresponding operations and movements in a simulated kitchen environment, where all objects to be manipulated are placed on a shelf in front of the mobile humanoid robot. In the Operational Space Task Trajectory Generalization and Generation Experiment, we applied the trajectory planning algorithm described in Section 3 to generate single-arm trajectory generalization, obstacle-avoidance trajectories, and coordinated dual-arm trajectories. In the Independent Dual-Arm Motion Experiment, the mobile humanoid robot moves, with its left and right arms each grasping objects from the shelf. The robot then turns to the right and moves to the target location, placing both objects at the designated positions on the table. In the Coordinated Dual-Arm Object Grasping Simulation Experiment, the right arm serves as the primary arm, and the left as the secondary arm. Both arms jointly grasp an object from the shelf, then turn and place the object at the target location on a distant table. In this case, the trajectory of the secondary arm is generated based on the motion trajectory of the primary arm.

### 5.1. Operational Space Task Trajectory Generalization and Generation Experiment

**Figure 9:**
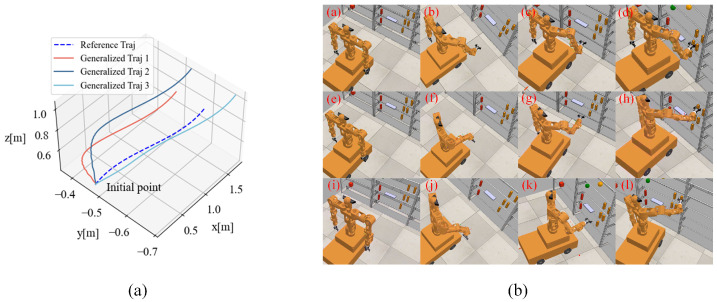
Trajectory generalization process of the robot’s right arm. (**a**) Generalized trajectory. (**b**) Execution process of the robot’s generalized trajectory. Where stages (a) to (d), (e) to (h), and (i) to (l) respectively indicate that the robot performs three different generalization trajectories.

In this section, to verify the adaptability and reliability of the operational space trajectory planning algorithm, we conducted an operational space trajectory generalization experiment. First, to evaluate the generalization effect of the trajectory, we used Dynamic Movement Primitives (DMP) to learn a reference trajectory for the right hand, with an initial position of (0.095 m, −0.466 m, 0.404 m) and a target position of (1.701 m, −0.586 m, 0.761 m). The DMP model can generate generalized trajectories similar to the reference trajectory’s characteristics based on changes in the target position, quickly creating corresponding trajectories for arm grasping tasks and ensuring that the trajectory consistently converges to the specified target position. Based on the different grasping positions of objects on the shelf, we generated three grasping trajectories. The trajectory generalization results are shown in Figure 9a. During trajectory execution, the end-effector of the mobile humanoid robot’s right arm follows the planned trajectory, and the whole-body coordinated motion is planned based on this trajectory. The motion process is shown in Figure 9b, with the three rows demonstrating the robot executing different generalized trajectories.

**Figure 10:**
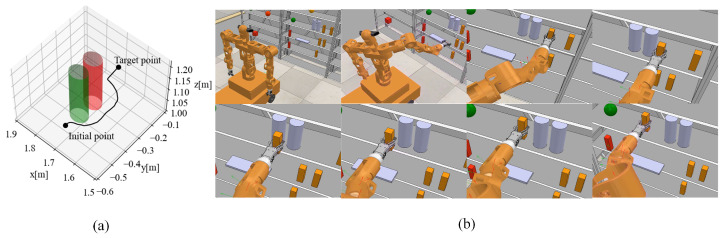
Obstacle avoidance process in operational space trajectory. (**a**) Obstacle avoidance trajectory. (**b**) Robot obstacle avoidance trajectory execution process.

We also designed a simulation experiment to verify the obstacle avoidance algorithm using Dynamic Movement Primitives (DMP). In the experiment, after the mobile humanoid robot’s right hand grasps the object, it performs an obstacle avoidance maneuver along the operational trajectory. Considering the object’s size and the gripper’s orientation, we reserved space around obstacles to generate an avoidance trajectory and prevent the object from colliding with obstacles. The specific experimental setup is shown in Figure 10b, where two cylindrical obstacles were placed on the shelf at positions (1.725 m, −0.4 m, 1.09 m) and (1.725 m, −0.3 m, 1.09 m), each with a radius of 0.04 m and a height of 0.2 m. The object’s starting position was (1.73 m, −0.54 m, 1.06 m), with a target placement position on the shelf of (1.73 m, −0.3 m, 1.09 m). Using the obstacle avoidance algorithm proposed in Section 3, the corresponding avoidance trajectory was planned, as shown in Figure 10a, where the trajectory diverges from the obstacle when approaching it. Figure 10b shows the mobile humanoid robot’s grasping and obstacle avoidance process. The experimental results demonstrate that the obstacle avoidance algorithm can generate effective avoidance trajectories with smooth trajectory variations.

At the same time, during the trajectory planning process, we evaluated the speed of trajectory generation. We generated the running trajectories for the same task three times (with 160 trajectory points each). The experimental results showed that the trajectory generation times for each run were 0.2252 s, 0.2444 s, and 0.2246 s, respectively. The average frequency of end-effector trajectory generation is thus calculated to be 695.76 Hz. This frequency is sufficient to support most trajectory planning processes. However, for more complex tasks or applications requiring higher precision, we will further optimize the algorithm to improve the trajectory generation efficiency.

### 5.2. Independent Arm Motion Experiment for Mobile Humanoid Robot

In this section, the end-effectors of the robot’s dual arms track pre-planned trajectories, with the previously described planning algorithm generating coordinated movements of the mobile humanoid robot’s upper limbs and mobile base to accomplish the task of turning and placing objects after grasping. Specifically, each arm grasps objects A and B from the shelf, and the robot then turns and moves to a distant target location to place the objects on the workbench table. The initial center position of the mobile humanoid robot’s base is at the origin of the world coordinate system. The initial grasping position of the left arm’s end-effector is (0.0955 m, 0.43966 m, 0.40401 m), and that of the right arm’s end-effector is (0.0955 m, −0.46596 m, 0.40401 m). On the shelf, the left arm’s grasping position for object A is (1.6761 m, 0.21825 m, 0.46133 m), and the right arm’s grasping position for object B is (1.701 m, −0.45675 m, 0.46133 m). On the target workbench, the left arm’s target placement position is (−2.05 m, −6.9 m, 1.05 m), and the right arm’s target placement position is (−2.475 m, −6.9 m, 1.05 m).

**Figure 11:**
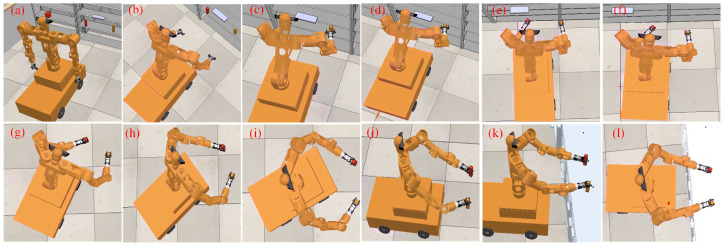
Independent dual-arm grasping and placement process. Stages (**a**–**l**) represent the turning and placing process after the robot grasps the object.

The entire motion process is shown in Figure 11. The mobile humanoid robot takes 5 s from the start of hand raising to grasping the objects, as illustrated in stages (a) to (d) in Figure 11. Upon reaching the target grasping position, both grippers take 3 s to secure the objects. After grasping, the mobile humanoid robot begins to turn toward the back-right direction and moves, following the end-effector trajectory, until the end-effectors reach the designated placement positions. The turning and placing process lasts 20 s, as shown in stages (e) to (l) in Figure 11.

**Figure 12:**
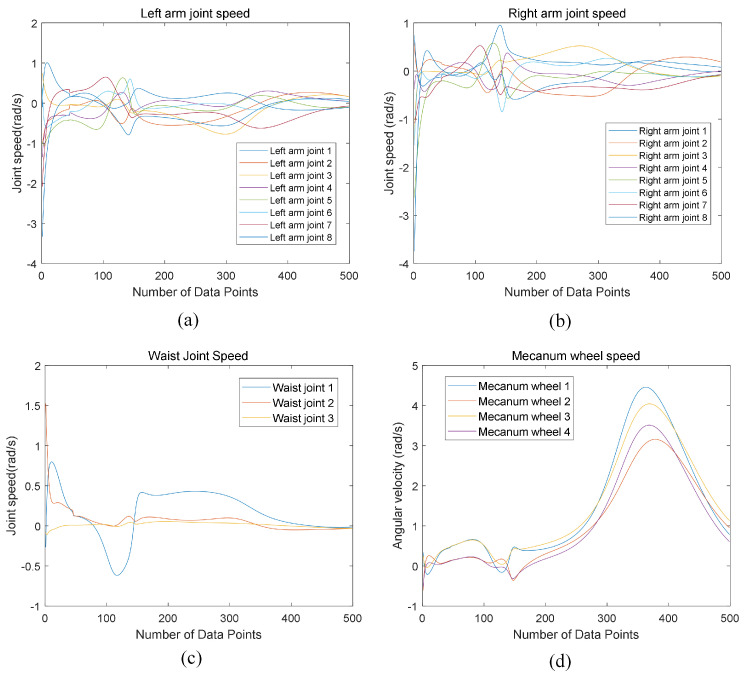
Joint velocity of the mobile humanoid robot. (**a**) Left arm joint velocity. (**b**) Right arm joint velocity. (**c**) Waist joint velocity. (**d**) Mecanum wheel velocity.

To analyze the motion process of arm-raising and object-grasping, Figure 12 presents the joint velocity changes over 5 s (with 500 data points) for the left arm joints, right arm joints, waist joints, and the Mecanum wheels on the mobile base of the humanoid robot, from the start of the hand-raising motion to object grasping. The simulation experiment and analysis of Figure 12 reveal that, when executing the specified end-effector trajectory task, the upper body joints (left arm, right arm, waist) of the mobile humanoid robot exhibit significant velocity spikes upon initiation. However, as the motion progresses, the joint velocities rapidly decrease, and subsequently, the velocity variations in each joint stabilize, indicating that the robot can achieve smooth motion control after initial acceleration.

**Figure 13:**
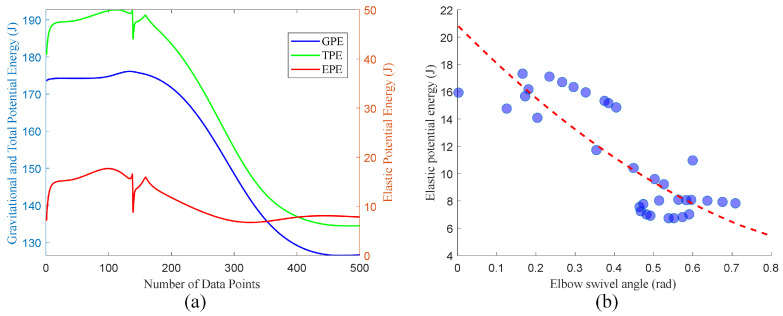
Energy variation diagram. (**a**) Total potential energy variation curve of the arm. (**b**) Trend of virtual torsional spring elastic potential energy of the arm. The blue dots represent the actual data, and the red dotted line represent the corresponding trend.

To validate our proposed objective of optimizing potential energy, we analyzed the potential energy changes of the right arm over 5 s (500 data points) during the initial phase of raising the arm to grasp the object. The initial height of the arm’s end-effector is 0.40 m, while the object’s grasping height is 0.46 m. Although the end-effector position of the entire arm continuously rises, the presence of the optimization term causes the gravitational potential energy of the entire arm to exhibit an overall downward trend after the movement starts, as shown in Figure 13a. The elastic potential energy is correlated with the arm angle; due to changes in wrist position and arm angle during the movement, the elastic potential energy fluctuates accordingly. However, as the task progresses, the gradient projection method guides the arm to move in the direction of decreasing total potential energy. During the arm-raising process to grasp the object, we calculated the values of the arm angle and elastic potential energy at corresponding positions. As the arm angle increases, the elastic potential energy tends to decrease, which aligns with the numerical trends in our gravitational potential energy expression, as shown in Figure 13b. Additionally, the arm angle is primarily within 0 to 0.8 rad, indicating a natural arm posture that facilitates prediction and assessment of the motion.

To evaluate the accuracy of the trajectory, we use common error metrics such as the Mean Squared Error (MSE) and Mean Absolute Error (MAE) to quantify the trajectory deviation. Using the right-hand trajectory of the robot bending down to grab an object from a shelf as an example, we analyze the MSE and MAE in the X-, Y-, and Z-directions for 500 3D trajectory points. Specifically, the MSEs in the X-, Y-, and Z-directions are $9.62\times 10^{-5}\;m^{2}$, $7.08\times 10^{-6}\;m^{2}$, and $1.30\times 10^{-4}\;m^{2}$, while the MAEs are 0.0093m, 0.0025m, and 0.0112m. Considering that the neural network’s output is not perfectly smooth and that motion interference occurs during the task, some error is introduced. At the same time, during the process of converting the end-effector trajectories to joint space angles, we calculated the corresponding decision speed. We performed three identical decision calculations (each involving 500 joint space trajectory points), and the results showed that the execution times for each run were 12.2579s, 12.3925s, and 12.3419s, respectively. We found that the execution time for each joint space trajectory point is mostly distributed between 0.020s and 0.0275s. The average frequency of decision generation was, therefore, calculated to be 40.55Hz. Considering the whole-body coordinated motion and the optimization of motion characteristics, the joint space trajectory generation speed is noticeably lower than the task space trajectory generation speed. This is because joint space computation involves more degrees of freedom and more complex calculations.

**Figure 14:**
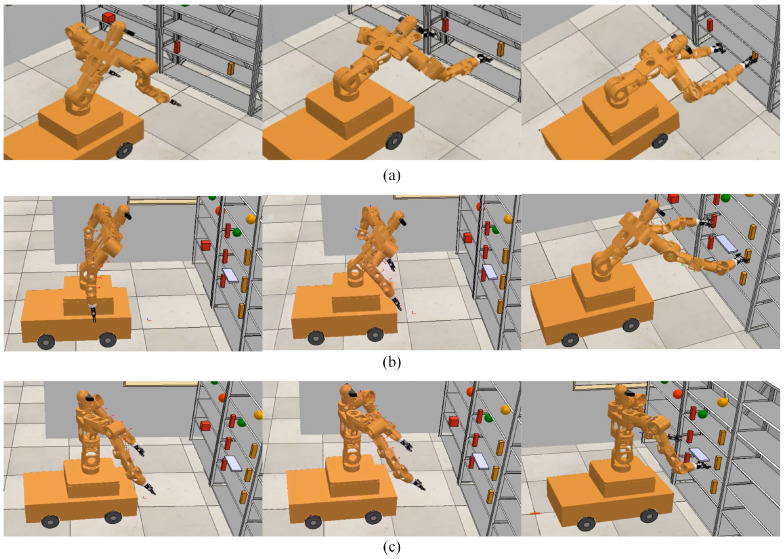
Comparison of our proposed method with the pseudo-inverse method. (**a**) Cases of motion planning failure using the pseudoinverse method. (**b**) Motion Effect of Pseudoinverse Method. (**c**) Motion Effect of the Proposed Method.

At the same time, we compared the motion effects, using the pseudo-inverse method as the benchmark for comparison. The pseudo-inverse method, as a classic approach for solving inverse kinematics, typically does not take into account the differentiated movements of different parts of the robot, especially in high-redundancy robots. This often results in an overall posture of the robot that does not meet expectations. Specifically, when using the pseudo-inverse method to control the entire robot, it fails to effectively distinguish the movements of the waist, arms, and chassis, leading to excessive bending of the waist, as shown in Figure 14b. In some extreme positions, a planning failure may occur (as shown in Figure 14a). This behavior is inconsistent with our expected human-like motion. In contrast, our proposed method, by introducing an optimization of potential energy, reduces the movement of the waist and aligns more closely with the principle of "more arm movement, less waist movement," as illustrated in Figure 14c.

In multiple grasping experiments, we recorded the computation time for 500 successful data points executed by the robot under pseudo-inverse control. The pseudo-inverse method’s single-step computation time ranges approximately from 0.002 to 0.006 s, with total computation times of 1.5868 s, 1.3799 s, and 1.4006 s, resulting in an average computation frequency of 343.46 Hz. In comparison, the computational frequency of our proposed algorithm is 40.55 Hz, which is relatively slower. To compare the motion accuracy, we calculated the MSE (Mean Squared Error) and MAE (Mean Absolute Error) between the actual end-effector positions and the reference trajectory for both the pseudo-inverse method and our approach in successful cases. The results (as shown in Table 2) indicate that there is little difference between our method and the pseudo-inverse method in these two metrics, with both methods completing the task within a low error range.

Through comparative analysis, we found that, while our method is slower than the classic pseudo-inverse method in terms of computational speed, it better aligns with the requirements of human-like motion in terms of movement quality. Our algorithm optimizes the potential energy control, coordinating the reduction of unnecessary waist movements during task execution, thereby enhancing the naturalness and stability of the motion. In future work, we plan to further optimize the algorithm structure to improve computational efficiency while maintaining accuracy.

### 5.3. Coordinated Dual-Arm Motion Experiment for Mobile Humanoid Robot

In this section, the robot uses the right arm as the primary arm and the left arm as the secondary arm, with the secondary arm’s trajectory generated based on the primary arm’s trajectory according to the algorithm in Section 2. The dual arms work together to grasp a panel, after which the robot turns and places the object on the workbench. Specifically, the robot first grasps panel C from the shelf with both arms, then turns and moves to a distant target location, placing the object on the workbench table. The initial state of the mobile humanoid robot is the same as in the independent dual-arm grasping task. The dimensions of panel C are (0.3 m, 0.1 m, 0.02 m), with its center located at (1.65 m, −0.125 m, 0.7 m) on the shelf. The final target placement position during the motion is at (−1.525 m, −6.9 m, 1.05 m).

**Figure 15:**
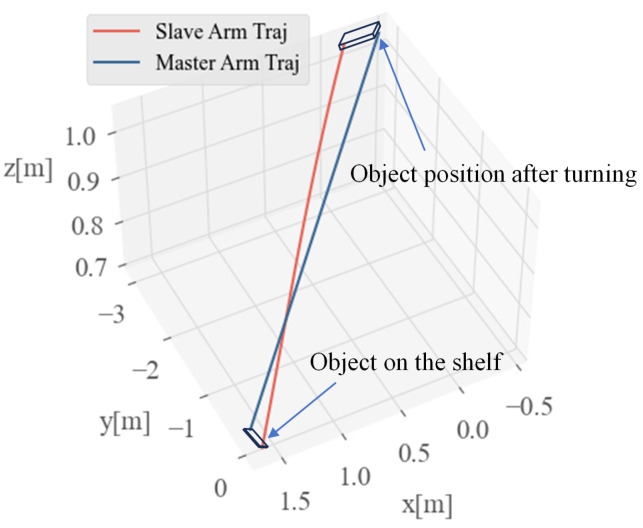
Illustration of primary-secondary arm task-space trajectories.

In this experiment, we captured the trajectory of the mobile humanoid robot completing the task of grasping an object from the shelf with both hands and turning within 10 s. Figure 15 shows the corresponding motion trajectories during this process, where the blue curve represents the planned task-space trajectory of the right arm (primary arm), and the red curve represents the task-space trajectory of the left arm (secondary arm) generated through the primary-secondary arm trajectory coordination algorithm described in Section 3. The figure also illustrates the position of the grasped object (0.3 m in length and 0.1 m in width). The experimental results indicate that the primary-secondary arm trajectory coordination algorithm can generate the secondary arm trajectory based on the primary arm trajectory and object dimensions successfully achieving coordinated dual-arm manipulation.

**Figure 16:**
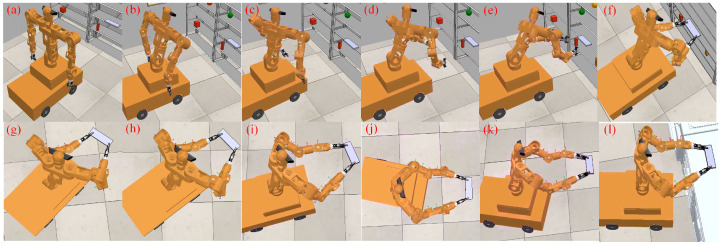
Dual-arm coordinated grasping motion process.

The entire motion process is shown in Figure 16. Stages (a) to (f) illustrate the approach phase, where both arms move toward the object. This phase lasts 5 s, with each arm’s trajectory planned independently. Once the arms grasp the object, the process enters the turning and placement phase, during which the arm trajectories are generated by the arm coordination algorithm. In this phase, the trajectory of the secondary arm is guided by the primary arm’s trajectory, lasting 20 s. Stages (f) to (l) demonstrate the motion of the left arm’s end-effector as it follows the trajectory generated for the primary arm using DMP, with the secondary arm’s motion adjusted according to the object’s dimensions. This validates the effectiveness of the dual-arm coordination algorithm.

**Figure 17:**
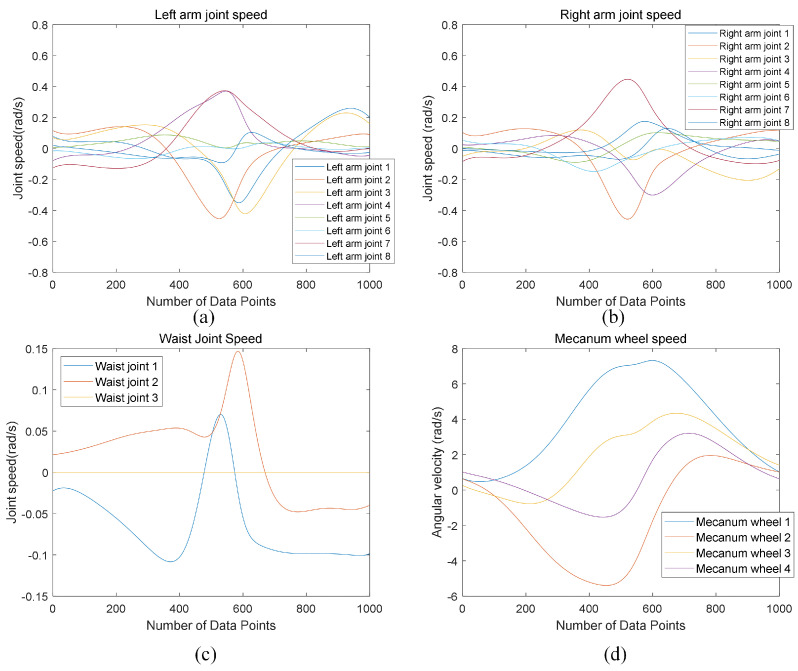
Joint velocity of the mobile humanoid robot. (**a**) Left arm joint velocity. (**b**) Right arm joint velocity. (**c**) Waist joint velocity. (**d**) Mecanum wheel velocity.

To analyze the dual-arm coordinated turning motion after grasping, Figure 17 shows the joint velocities of the left arm, right arm, waist, and Mecanum wheels on the mobile base over a 10 s period during the turning process. The trajectory planning algorithm for the mobile humanoid robot’s primary and secondary arms enables smooth motion control, ensuring coordinated dual-arm grasping of the object.

Based on the experiments of independent dual-arm object grasping and coordinated dual-arm object grasping by the mobile humanoid robot, the proposed algorithm enables coordinated control of all 23 degrees of freedom in the robot according to the planned end-effector trajectories. The motion strategy, based on optimizing the arm gravitational potential energy and virtual elastic potential energy, allows the robot’s arms to generate human-like movements while reducing the gravitational and elastic potential energy. Additionally, the two trajectory generation modes—independent dual-arm motion and coordinated dual-arm motion—enable the mobile humanoid robot to accomplish various movement tasks effectively.

## 6. Discussion

The practical significance of this research lies in the fact that the proposed whole-body coordinated motion control method can enable humanoid robots with high redundancy in degrees of freedom to implement human-like control strategies. By mimicking human movement characteristics, the robot can perform more predictable and human-like actions. This manuscript primarily focuses on the design of the humanoid robot’s full-body controller and includes basic trajectory planning. As the system continues to evolve and integrate, we will implement upper-level planning to predict and adjust movement trajectories based on real-time feedback from moving objects, aiming for more accurate control and collaboration in the future.

Our method explores the relevant arm movement characteristics, which exhibit high adaptability in robotic arms. Considering current robots, whether simple robotic arms or humanoid robots, regardless of the number of degrees of freedom in the arms, they essentially consist of two parts: the upper arm and the forearm. Both parts possess corresponding gravitational potential energy, and during operation, the upper arm and forearm form an arm plane. To adjust the posture of the entire arm, we use the arm shape angle as a universal variable for the torsion angle of the virtual torsion spring, which allows the potential energy term to be applicable to robotic arms or humanoid robots with different degrees of freedom. Additionally, we generate the end-effector trajectory using an algorithm based on dynamic motion primitives, which does not consider degrees of freedom when generating the trajectory. Therefore, our method demonstrates adaptability across different degrees of freedom.

Currently, our algorithm has primarily been validated in a simulation environment. However, simulation environments cannot fully replicate the complexities of the real world, such as sensor noise, mechanical gaps, friction, limited positioning accuracy, and constantly changing environmental conditions. Future research will focus on enhancing the robustness of the perception system in the real world, such as using sensor fusion and filtering algorithms to reduce the impact of noise on motion control. Additionally, a more refined feedback mechanism will be introduced, allowing the robot to adjust its motion trajectory based on real-time feedback, enabling it to better handle the challenges of the actual environment.

In future research, we plan to expand the experimental scope to test the virtual torsional spring stiffness parameters under different robot configurations (such as varying arm lengths, mass distributions, or joint constraints) in order to validate the adaptability of the method and ensure the universality of the potential energy optimization approach.

## 7. Conclusions

In this study, by collecting and analyzing human arm motion data and applying human-robot posture mapping, we derived corresponding robot postures for human postures. Based on this, we constructed gravitational potential energy and virtual elastic potential energy models for the robot arm, using a neural network to fit the variable stiffness of the arm’s virtual torsional spring, thereby describing relevant motion characteristic metrics. By integrating the designed operational space trajectory and motion characteristic metrics of the mobile humanoid robot, we achieved human-like coordinated control across the robot’s 23 degrees of freedom through a whole-body motion controller. In future work, we will further explore human motion planning principles to realize even more human-like coordinated motion control for mobile humanoid robots.

## Figures and Tables

**Table 1:** Kinematic parameters of the mobile humanoid robot.

Joint (i)	$\alpha_{i-1}$ (rad)	$a_{L_{i-1}/R_{i-1}}$ (mm)	$d_{L_{i-1}/R_{i-1}}$ (mm)	$\theta_{L_{i-1}/R_{i-1}}$ (rad)
1	− 1.5708	0	0	3.1416
2	−1.5708	0	377	−1.5708
3	−1.5708	0	34.5	3.1416
4/12	−1.5708	144.5/−144.5	130/−130	−1.5708
5/13	1.5708	0	−322.5/322.5	1.5708
6/14	1.5708	0	0	1.5708
7/15	1.5708	0	−266.2	1.5708
8/16	1.5708	0	0	0
9/17	−1.5708	0	−248.2	0
10/18	1.5708	0	0	0
11/19	−1.5708	0	−336.1	3.1416

**Table 2:** Comparison between pseudo-inverse method and our proposed method.

Comparison Metrics	Pseudo-Inverse Method	Our Proposed Method
Execution Frequency (Hz)	343.46	40.55
MSE of Trajectory in X-Direction (m^2^)	$1.02\times 10^{-4}$	$9.62\times 10^{-5}$
MSE of Trajectory in Y-Direction (m^2^)	$1.07\times 10^{-5}$	$7.08\times 10^{-6}$
MSE of Trajectory in Z-Direction (m^2^)	$9.85\times 10^{-5}$	$1.30\times 10^{-4}$
MAE of Trajectory in X-Direction (m)	0.0096	0.0093
MAE of Trajectory in Y-Direction (m)	0.0030	0.0025
MAE of Trajectory in Z-Direction (m)	0.0097	0.0112

## Data Availability

The data presented in this study are available on request.

## References

[1] Xu Y., Chen Z., Deng C., Wang S., Wang J. (2024). LCDL: Toward Dynamic Localization for Autonomous Landing of Unmanned Aerial Vehicle Based on LiDAR–Camera Fusion. IEEE Sens. J..

[2] Deng C., Wang S., Wang J., Xu Y., Chen Z. (2024). LiDAR Depth Cluster Active Detection and Localization for a UAV with Partial Information Loss in GNSS. Unmanned Syst..

[3] Fan X., Shu X., Tu B., Liu C.Y., Ni F., Jiang Z. (2023). A humanoid robot teleoperation approach based on waist-arm coordination. Ind. Robot.

[4] Dragan A.D., Srinivasa S.S. Familiarization to Robot Motion. Proceedings of the 2014 9th ACM/IEEE International Conference on Human-Robot Interaction (HRI).

[5] Kulić D., Venture G., Yamane K., Demircan E., Mizuuchi I., Mombaur K.D. (2016). Anthropomorphic Movement Analysis and Synthesis: A Survey of Methods and Applications. IEEE Trans. Robot..

[6] Su H., Qi W., Hu Y., Karimi H.R., Ferrigno G., Momi E.D. (2020). An Incremental Learning Framework for Human-Like Redundancy Optimization of Anthropomorphic Manipulators. IEEE Trans. Ind. Inform..

[7] Ferrer-Ballester M.A., Díaz M., Quintana J.J., Carmona-Duarte C., Plamondon R. (2023). Extending the kinematic theory of rapid movements with new primitives. Pattern Recognit. Lett..

[8] Taniai Y., Naniwa T., Nishii J. (2022). Optimal reaching trajectories based on feedforward control. Biol. Cybern..

[9] Young S.J., Pratt J., Chau T. (2009). Target-Directed Movements at a Comfortable Pace: Movement Duration and Fitts’s Law. J. Mot. Behav..

[10] Zhao J., Xie B., Song C. (2014). Generating human-like movements for robotic arms. Mech. Mach. Theory.

[11] Li M., Guo W., Lin R., Wu C., Han L. (2018). An Efficient Motion Generation Method for Redundant Humanoid Robot Arm Based on the Intrinsic Principles of Human Arm Motion. Int. J. Humanoid Robot..

[12] Qu J., Zhang F., Wang Y., Fu Y. (2019). Human-like coordination motion learning for a redundant dual-arm robot. Robot. Comput. Integr. Manuf..

[13] Yang C., Zeng C., Fang C., He W., Li Z. (2018). A DMPs-Based Framework for Robot Learning and Generalization of Humanlike Variable Impedance Skills. IEEE/Asme Trans. Mechatron..

[14] Chen F., Wang F., Dong Y., Yong Q., Yang X., Zheng L., Gao Y., Su H. (2023). Sensor Fusion-Based Anthropomorphic Control of a Robotic Arm. Bioengineering.

[15] Duarte N.F., Raković M., Santos-Victor J. Biologically Inspired Controller of Human Action Behaviour for a Humanoid Robot in a Dyadic Scenario. Proceedings of the IEEE EUROCON 2019—18th International Conference on Smart Technologies.

[16] He C., Xu X., Zheng X.F., Xiong C., Li Q., Chen W., Sun B.Y. (2021). Anthropomorphic Reaching Movement Generating Method for Human-Like Upper Limb Robot. IEEE Trans. Cybern..

[17] Kuo P.H., Hu J., Lin S.T., Hsu P.W. (2022). Fuzzy Deep Deterministic Policy Gradient-Based Motion Controller for Humanoid Robot. Int. J. Fuzzy Syst..

[18] Pignat E., Calinon S. (2017). Learning adaptive dressing assistance from human demonstration. Robot. Auton. Syst..

[19] Yi J.B., Kim J., Kang T., Song D., Park J., Yi S.J. (2022). Anthropomorphic Grasping of Complex-Shaped Objects Using Imitation Learning. Appl. Sci..

[20] Yang A., Chen Y., Naeem W., Fei M., Chen L. (2021). Humanoid motion planning of robotic arm based on human arm action feature and reinforcement learning. Mechatronics.

[21] Artemiadis P.K., Katsiaris P.T., Kyriakopoulos K.J. (2010). A biomimetic approach to inverse kinematics for a redundant robot arm. Auton. Robot..

[22] Gäbert C., Kaden S., Thomas U. Generation of Human-like Arm Motions using Sampling-based Motion Planning. Proceedings of the 2021 IEEE/RSJ International Conference on Intelligent Robots and Systems (IROS).

[23] Ginesi M., Meli D., Roberti A., Sansonetto N., Fiorini P. (2020). Dynamic Movement Primitives: Volumetric Obstacle Avoidance Using Dynamic Potential Functions. J. Intell. Robot. Syst..

[24] Corke P., Haviland J. Not your grandmother’s toolbox—The Robotics Toolbox reinvented for Python. Proceedings of the 2021 IEEE International Conference on Robotics and Automation (ICRA).

